# Histopathological Comparison between Bone Marrow- and Periodontium-derived Stem Cells for Bone Regeneration in Rabbit Calvaria

**Published:** 2016-02-01

**Authors:** Z. Kadkhoda, A. Safarpour, F. Azmoodeh, S. Adibi, A. Khoshzaban, N. Bahrami

**Affiliations:** 1 Periodontology Department, Dental Faculty of Tehran University of Medical Sciences, Tehran, Iran; 2 Pathology Department, Imam Khomeini Hospital, Tehran University of Medical Sciences, Tehran, Iran; 3 Dental Research of Torabinejad Research Centre, Isfahan, Iran; 4 Stem Cells Preparation Unit, Farabi Eye Hospital, Tehran University of Medical Sciences, Tehran, Iran; 5 Oral and Maxillofacial Surgery Department, School of Dentistry, Tehran University of Medical Sciences, Tehran, Iran; 6 Craniomaxillofacial Research Center, Tehran University of Medical Sciences, Tehran, Iran; 7 *Iranian Tissue Bank and Research Center, Tehran University of Medical Sciences, Tehran, Iran*

**Keywords:** Stem cell, Periodontium, Osteoblasts, Bone regeneration, Periodontitis, Periodontal ligament, Mouth diseases, Mesenchymal stem cells, Cell- and tissue-based therapy, Cell Lineage

## Abstract

**Background:**

Periodontitis is an important oral disease. Stem cell therapy has found its way in treatment of many diseases.

**Objective:**

To evaluate the regenerative potential of periodontal ligament-derived stem cells (PDLSCs) and osteoblast differentiated from PDLSC in comparison with bone marrow-derived mesenchymal stem cells (BM-MSCs) and pre-osteoblasts in calvarial defects.

**Methods:**

After proving the existence of surface markers by flow cytometry, BM-MSCs were differentiated into osteoblasts. 5 defects were made on rabbit calvaria. 3 of them were first covered with collagen membrane and then with BM-MSCs, PDLSCs, and pre-osteoblasts. The 4^th^ defect was filled with collagen membrane and the 5^th^ one was served as control. After 4 weeks, histological (quantitative) and histomorphological (qualitative) surveys were performed.

**Results:**

Both cell lineages were positive for CD-90 cell marker, which was specifically related to stem cells. Alizarin red staining was done for showing mineral material. RT-PCR set up for the expression of Cbfa1 gene, BMP4 gene, and PGLAP gene, confirmed osteoblast differentiation. The findings indicated that although PDLSCs and pre-osteoblasts could be used for bone regeneration, the rate of regeneration in BM-MSCs-treated cavities was more significant (p<0.0001).

**Conclusion:**

The obtained results are probably attributable to the effective micro-environmental signals caused by different bone types and the rate of cell maturation.

## INTRODUCTION

One of the important oral diseases is periodontitis. It is an acute inflammatory disease [1], which is caused by microorganisms that adhere to and grow on the tooth’s surfaces [1]. In this disease, periodontal ligament (PDL) and alveolar bone are involved. The symptoms include gingival inflammation (GI) and periodontal attachment loss, which gives rise to bone loss around the teeth [2]. With regard to the costs and the problems associated with this disease, finding a suitable treatment and replacing it with routine treatments is inevitable. One of the replaced treatments is regenerative treatment based on applying cells, especially stem cells. It has already been determined that for regenerative treatments such as guided tissue regeneration (GTR), the existence of growth factors plays a significant role [3]. The following factors play a vital role in tissue regeneration: cells, scaffolds and signaling molecules. Although scaffolds and signaling molecules are clinically more identified, cells can act more differently. Collagen membrane guides regeneration and provides favorable conditions for cell growth and tissue reconstruction. As the available commercial scaffolds often do not have induction capacity, it seems that using growth factors with scaffolds can expedite the regenerative process while the cells are the main factors in this procedure [2, 4, 5]. These cells should be non-immunologic and have high regenerative potential while they are easily cultured and have the capacity to differentiate into different cell types [6]. Stem cells are included in these cell types and their existence in different parts of the body, such as bone marrow, cornea, and skin is confirmed [7]. In regeneration treatments, bone marrow-derived mesenchymal stem cells (BM-MSCs) are used, which can differentiate into osteoblasts, chondrocytes, adipocytes, and neuromuscular cells *in vivo* and *in vitro* [8]. Another tissue considered for regeneration treatment is PDL. Periodontium consists of two parts: “bone and cementum,” and “gingiva and PDL” [9]. Periodontitis causes tissue injury to the periodontium; PDL can improve periodontium because this cell type plays an important role in nourishing teeth, homeostasis, and regeneration of injured tissue. This feature may be attributed to the existence of progenitor cells [2, 9]. PDL is a connective tissue between alveolar bone and cementum. It has a heterogeneous cell population, including fibroblasts and progenitor cells that can differentiate into osteoblasts and cementoblasts, which have the characteristics of osteoblasts. It has recently been demonstrated that PDL tissue is consisted of mesenchymal stem cells (MSCs), which are distinguished from other cells by detecting their surface markers [10]. Considering the development of regeneration treatment and need for easy and rapid access to a suitable cell source in this kind of treatment, the objective of the current work was to isolate the autologous stem cells from bone marrow and periodontal ligament (BM-MSCs and PDLSCs), evaluate their differentiation potential into osteoblasts, and compare regenerative potential between these two types of stem cells in an animal model. Also, the reconstruction rate of calvaria using the PDLSCs-differentiated osteoblasts was compared to the stem cell-treated groups.

## MATERIALS AND METHODS

Isolation and Culture of Adult Stem Cells

**Figure 1:**
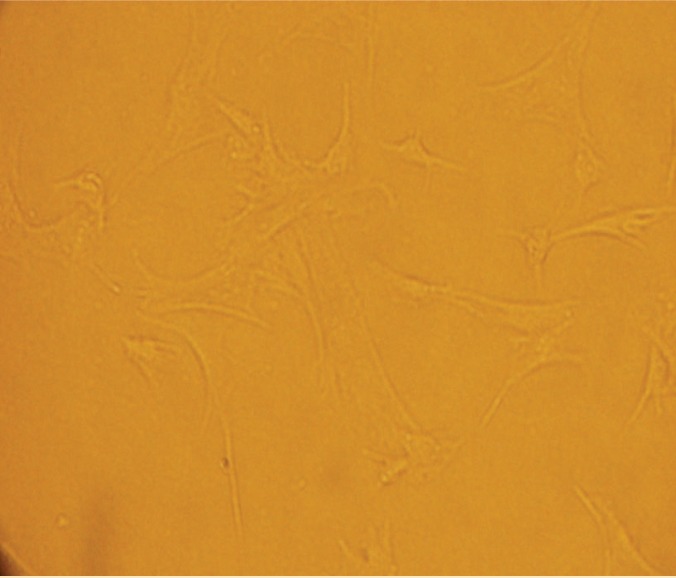
Microscopic view of MSCs

The study was performed on 10 adult New Zealand white rabbits weighing 2.5 kg purchased from Pasteur Institute of Iran. The rabbits were maintained under 12:12 h light:dark cycle and kept in special cages with access to water and pellets *ad libitum*. Bone marrow was obtained from tibia bone by aspiration and the periodontal tissue from the surfaces of two mandibular central incisors. For red blood cell (RBC) lysis, physiological saline-Ficoll solution was added to the sample. Then, the sample was rinsed twice with sterile phosphate buffer saline (PBS) and was centrifuged at 1000 rpm for 30 min (Eppendorf Centrifuge). After centrifuging, DMEM solution (Dulbcco’s Modified Eagle Medium, Sigma, USA), containing 10% fetal bovine serum (FBS), 1% unessential amino acids, 100 ng/mL sodium pyruvate (Gibco, USA), 100 units penicillin, and 100 mg/mL streptomycin, was used for the remaining cells in a 75-cm^2^ flask. Then, 100 ng dexamethasone was added and the culture medium was changed after 24 h and then every 4 days. When 4/5 of the bottom of the flask covered with cells, the cell passage was done using 0.25% trypsin-EDTA solution. The cells incubated at 37 °C with 5% CO_2_.(Fig 1).

Isolation and Culture of PDLSCs

Periodontium was separated from the root with a curette. The sample was cut into several sections and then were washed twice in DMEM culture medium (Sigma, USA). To isolate PDLSCs from the adjacent tissue, the sections were placed in collagenase type I solution and were enzymatically digested for 1 h at 37 °C using 4 mg/mL dispase. The cell suspension (10^5^ cells) consisting of DMEM low glucose (Sigma, USA), 10% FBS, glutamine-L, 100 units penicillin, 100 mg/mL streptomycin, 5 mg/mL amphotrypcine B, and 0.05% EDTA, was passaged in a 25-cm^2^ flask.

Assessment of Stem Cells by Flow Cytometry

After the second passage, MSCs were prepared for flow cytometry to investigate the cell population and the final isolation of stem cells. The cells were treated and counted using 0.25% trypsin-EDTA. Then, 10^5^–10^6^ cells were incubated on a rocker rotator and were centrifuged at 1000 rpm for 6 min. The next stage was to add 2 units of 3% FBS to make the culture medium suitable for adding monoclonal antibody. It was then placed at room temperature for 30 min. Again, they were centrifuged at 1000 rpm for 6 min and PBS was added to the solution. The cell mixture was passed through a nylon mesh and again PBS with anti-CD166, anti-CD105, anti-CD45, anti-CD34, anti-CD73, anti-CD44, anti-CD90 (FITC), and CD146 were added to it. The resulting mixture was placed at 4 °C out of light for 45 min. 

Differentiation of PDLSCs into Osteoblasts 

After the isolation of PDLSCs, they were placed in DMEM plus 10% FBS, including 10 mM β-glycerol phosphate, 10^-7^ M dexamethasone, and 50 mg/mL ascorbic acid. To analyze osteoblast differentiation, alizarin red staining technique was used as a qualitative analysis. Then, the samples were observed under a digital inverted microscope (Cetti, Spain).

Alizarin Red Staining

In this technique, cell culture medium was washed with Tyrode solution and PBS, and then fixed with 4% paraformaldehyde. The cells were stained using 40 Mm alizarin red for 10 min at room temperature and rinsed with water and PBS.

DAPI Staining

DAPI is a popular nuclear staining, which is used in multicolor florescent techniques. Its blue florescence is in clear contrast with green, yellow, or red florescent probes of other structures. DAPI stains nuclei specifically with no or little cytoplasmic effect. The following protocol was applied for staining of tissue sections or for cultured cells on glass slides. For preparation of DAPI stock solution (14.3 mM or 15 mg/mL), 10 mg DAPI (molecular probes) (Introversion, Grand Island, USA) was mixed with 2 mL diphenylformamide (DMF) to dissolve, aliqouted and kept at -20 °C. The DAPI working solution (100 ng/mL or 300 nM in PBS), containing 2 µL DAPI stock solution and 100 mL PBS, was stored at 4 °C in a brown or aluminum foil-wrapped bottle to protect it from light. These two sections were incubated for 30 min at room temperature in dark. 

RT-PCR

RT-PCR was performed for analyzing and confirming the differentiation of PDLSCs into osteoblasts. In this analysis, the expression of Cbfa1 gene, BMP4 gene, and BGLAP gene was analyzed as osteoblast-specific genes. DLX3 gene plays a significant role in detecting PDLSCs. Consequently, it was investigated to prove the existence of PDLSCs. It should be noted that it was negative for BM-MSCs and positive for PDLSCs. Moreover, GAPDH gene expression was considered “the positive control” in all cells. The primers used were as follows:

Fw (GAPDH): 5’ AAA TTG AGC CCG CAG CCT 3’

Rev (GAPDH): 5’ GGG TTG AGC ACA GGG TAC TTT A 3’

Fw (BGLAP): 5’ AGC CAC CGA GAC ACC ATG AGA 3’

Rev (BGLAP): 5’ TTT TCA GAT TCC TCT TCT GGA G 3’

Fw (BMP4): 5’ GCC GGG GAA GAG GAG GAG 3’

Rev (BMP4): 5’ CAA TAT GGT CAA AAC ATT TGC 3’

Fw (Cbfal): 5’ ATG CTT CAT TCG CCT CAC AAA 3’

Rev (Cbfal): 5’ AAG CTT TGC TGC TGA CAC GGT GTC 3’

Fw (Dlx3): 5’CTA CCG GCA ATA CGG GGC GT 3’

Rev (Dlx3)**:** 5’AGT GGA GTG GGA AGA GGT GTC CCA 3’

According to the manufacturer’s recommendations, all RNA was extracted using RBC kit (*www.Real-biotech.com*). For gene amplification, mi-RT GO kit (*www.mymetabion.com*) was utilized. The cDNA preparation process was continuously carried out in a thermocycler. PCR procedure was performed using 25 µL of reaction solution, containing 3 µL mRNA, 8.5 µL of DNase/RNase-free water, 1 µL RNase enzyme, 4 µL inverted primer, 2 µL forward primer, 25 µL dNTP mixture, and 5 µL buffer.

Cell Implantation into Rabbit Calvaria 

To investigate the activation rate of BM-MSCs and PDLSCs in reconstructing and regenerating bone defects, and also compare it with their self-renewing potential resulted from the bone induction around the bone defects, five through-and-through defects were made on the frontal and parietal bones of each rabbit using a trephine bur. Three cavities were first covered with the collagen membrane (Bio-Guide, Switzerland). Then, cell suspensions of 2×10^7^ BM-MSCs in the first cavity, 10^7^ PDLSCs in the second cavity, and 3-day pre-osteoblasts in the third cavity were separately placed on this membrane. The fourth cavity was covered with collagen gel and filled with DMEM. The fifth cavity served as “control” (empty). The skull was separated after sacrificing the animal with intracardiac injection of 10% ketamine and prepared for histomorphometric studies.

Preparing Histological Samples

The skull was kept in 25% nitric acid for 72 h to separate the soft tissues of the calvaria from the bony part. The bone sections, including superior orbital rim, the frontal, and parietal bones, were embedded in 10% formalin. Ten days after fixing, they were placed in paraffin and cut into 5-µm sections. The samples were stained with hematoxylin and eosin (H&E) and investigated under light microscope (Olympus, USA).

Histomorphological Evaluation 

In histological evaluation, the bone regeneration was vitally performed, whereas the regenerated bone was connected to its adjacent bone without connective tissue. Five defects were surgically produced in each rabbit calvarium. Twenty glass slides were prepared for each defect. Overall, there were 1000 slides for histopathological evaluation. Friedman test was applied to analyze inflammation and bone regeneration; pairwise comparison was performed for collagen gel and control groups. 

## RESULTS

Flow Cytometry

**Figure 2:**
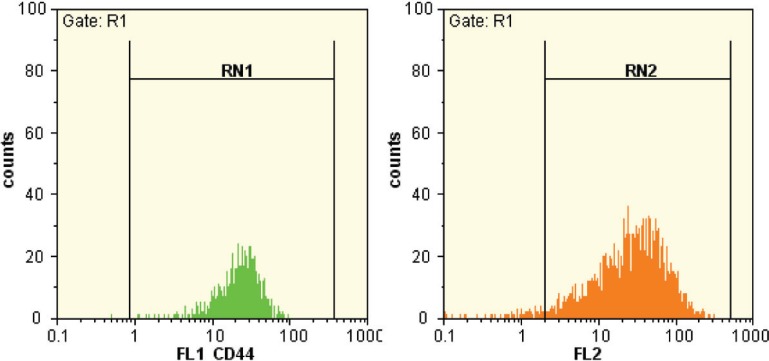
Diagram of CD44 flowcytometry

In flow cytometry, passage 2 cells were evaluated for expression of cell surface markers; the results indicated successful isolation of BM-MSCs and PDLSCs. Both cell types were positive for CD90, CD105, CD73, and CD166 cell markers, which are specifically related to stem cells; the cells were however, negative for CD45 and CD34. Almost all (96.96%) of PDLSCs and 99.72% for BM-MSCs expressed CD44 surface markers (Fig 2). More than half (59.49%) of PDLSCs and 32.80% of BM-MSCs expressed CD90 surface marker. CD146 was found positive in periodontium, but it was not expressed in BMSCs. In fact, it is not a specific marker; it is distinctive.

Stem Cells Differentiation into Osteoblasts

**Figure 3:**
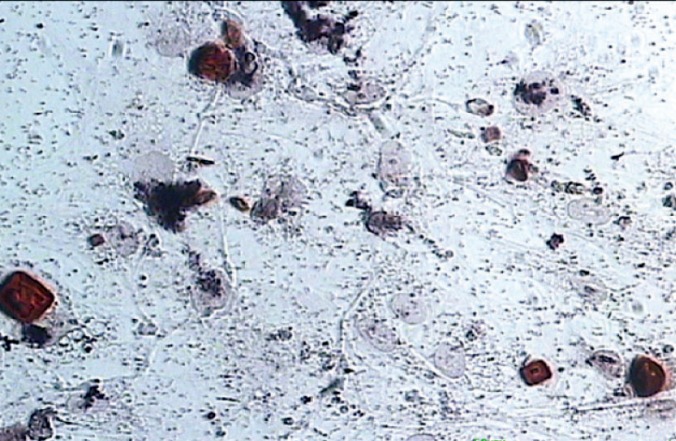
Alizarine staining

By adding differential cytokines, the stem cells were differentiated into osteoblasts. Calcium secretion was shown by alizarin red staining. Calcium deposition was detected by appearing a red-orange color (Fig 3).

DAPI Staining

The stem cell nuclei were stained with DAPI (blue) and the results proved the osteogenic activity of the cells.

RT-PCR

**Figure 4:**
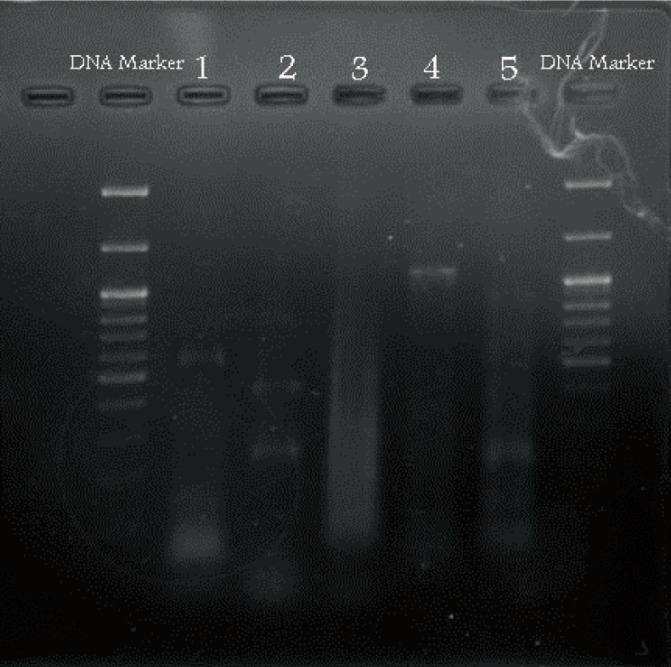
Gel electrophoresis. Lanes 1, 2, 3, 4, and 5, indicate GAPDH mRNA, BGLAP mRNA, BMP4 mRNA, and Cbf1 mRNA, respectively

To confirm differentiation into osteoblasts, osteoblast-specific gene expression was measured. After regeneration and differentiation, 1.5% agarose gel electrophoresis and ethidium bromide staining were applied. The quantitative measurement was done by fermentas DNA marker (Fig 4). For confirming the existence of human PDLSCs, this gene should be positive because DLX3 increases during periodontium formation in the tooth bud. In the current study, this gene was positive for PDLSCs.

Histomorphological Evaluation 

**Figure 5:**
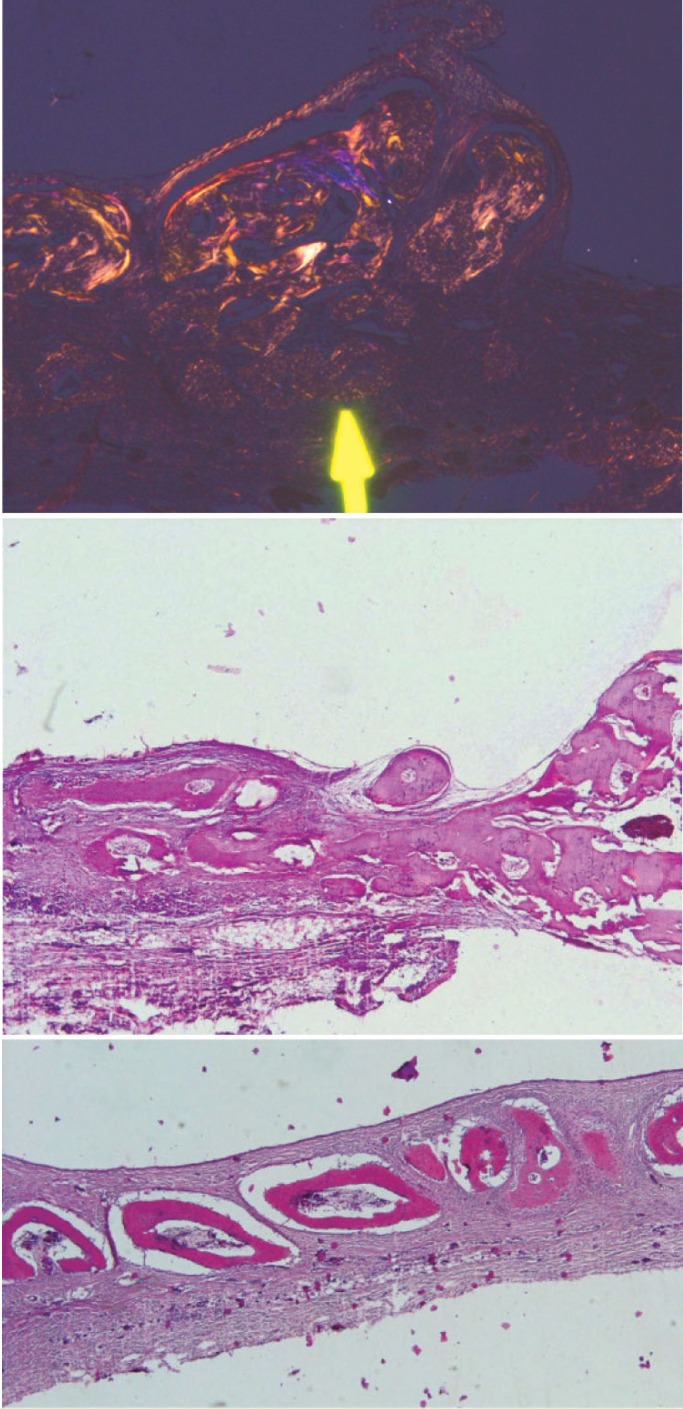
Photomicrograph of BMSCs

**Figure 6:**
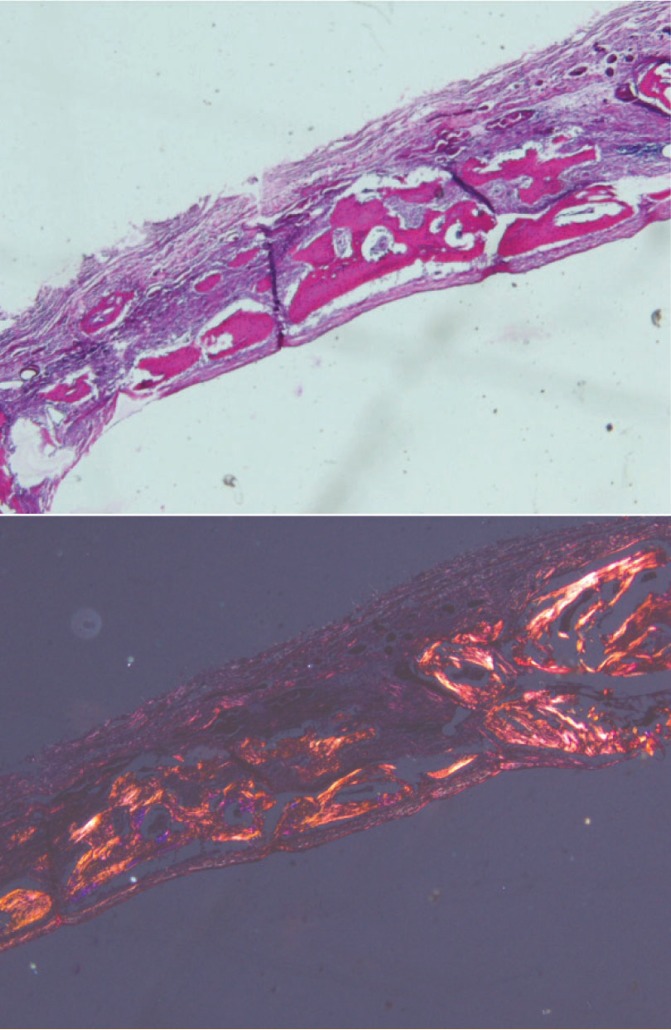
Photomicrograph of PDLSCs

**Figure 7:**
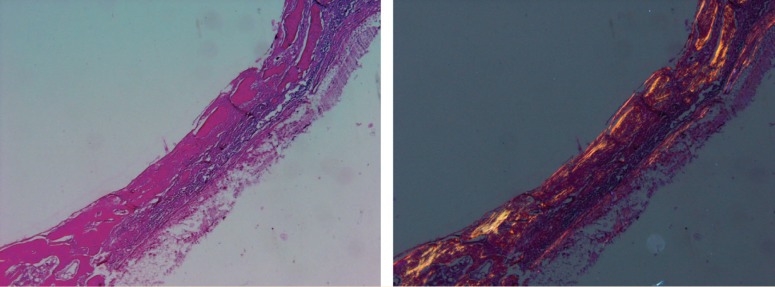
Photomicrograph of pre-osteoblast

**Figure 8:**
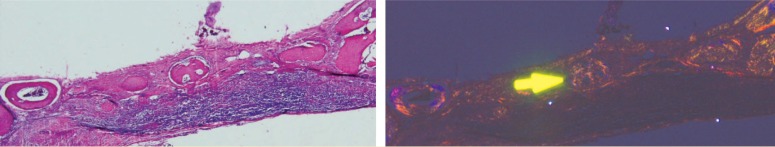
Photomicrograph of membrane

**Figure 9:**
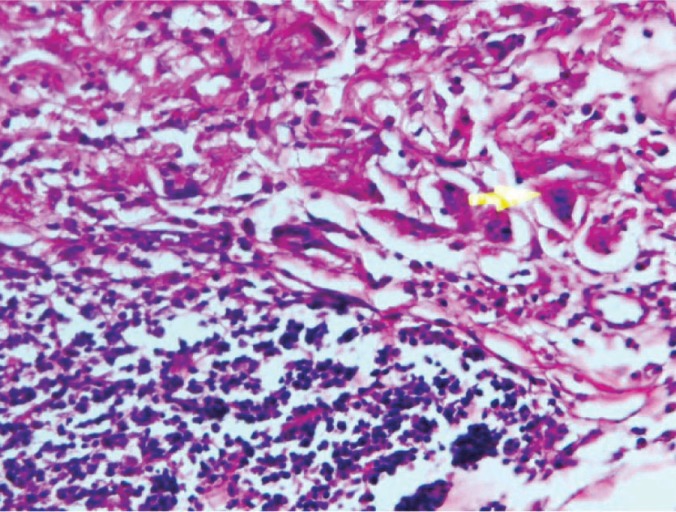
Photomicrograph of the control group

**Figure 10:**
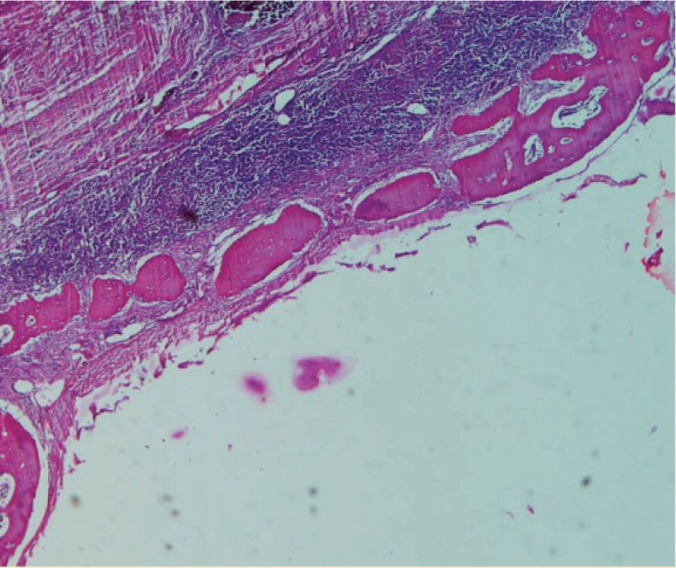
Photomicrograph of collagenous membrane

In all investigated groups, the studied variables, consisted of inflammation and bone regeneration, showed significant changes (p<0.0001). After four weeks, there were no significant differences between three of five groups in which the bone had been regenerated by BM-MSCs, PDLSCs, and pre-osteoblasts. However, in comparison to the other two groups treated with pure collagen gel and control, a significant difference was seen in the rate of bone regeneration (Figs 5-10).

Pairwise comparison of inflammation and new bone formation among the five studied groups showed a significant difference in comparison to the control group. Regarding the percentage of new bone formation in BMSCs-treated group, there was a significant difference between this group and PDLSCs-treated (p=0.018), collagen membrane-treated (p=0.001), and the control (p=0.001) groups. No significant difference was observed in the rate of bone reconstruction between BMSCs- and pre-osteoblast-treated groups (p=0.626).

With regard to the percentage of newly formed bone in pre-osteoblast-treated group, there was no significant difference in comparison with PDLSCs-treated (p=1.000), BMSCs-treated (p=0.626), collagen membrane-treated (p=1.000), and the control (p=1.000) groups.

Considering the percentage of newly formed bone in collagen membrane-treated group, there was a significant difference between this group and PDLSCs-treated (p<0.001), and BMSCs-treated (p=0.001) groups. No significant difference was observed between this group and pre-osteoblast-treated (p=1.000) and the control (p=1.000) groups. The percentage of newly formed bone in control group indicated a significant difference in comparison with PDLSCs-treated (p=0.003), and BMSCs-treated (p<0.001) groups. No significant difference was observed between this group and pre-osteoblast-treated (p=1.000) and the control (p=1.000) groups.

## DISCUSSION

Periodontitis involves the inflammation of supportive tissue (alveolar bone, cementum, and gingiva) around the teeth, alveolar bone loss, and subsequent loss of teeth [1, 2]. Sharpey’s fibers, which are the strong collagenous fibers and insert into the cementum and into the periosteum of the alveolar bone, are degenerated and their reconstruction is not autonomously occurred [5, 6]. This is the leading cause of periodontitis. Due to the complexity of periodontal tissue, the regeneration of soft and hard tissues of periodontium was studied. Although some treatments such as GBR/GTR and bone graft were performed on animal and human models, the results were not acceptable [6]. Recent studies have indicated that a stromal cell population exists in periodontal tissue, which can regenerate bone, cementum, and PDL [3, 9]. Most of the progenitor cells have been detected in mouse PDL [2, 10]. Endosteal spaces of alveolar bone are the source of this cell type and they gradually migrate to PDL area [9, 10]. With regard to cell kinetics and the observation of cell morphology, these progenitor cells were considered as stem cells [10]. It appeared that PDL is consisted of a heterogeneous population of cells that not only have the differentiation potential into cementoblasts but also they can differentiate into adipocytes and osteoblasts under *in vitro* conditions and into cementum- and PDL-like tissues under *in vivo* conditions [3, 10]. Considering the above-mentioned morphological and physiological features and the differentiation potential of these cells into different cell types [7, 8], this cell population is considered as stem cells. PDLSCs are on the surface of the root (apical and coronal). Periodontitis causes PDL destruction, whereas the stem cells and the surface of the root remain in the apical area and PDL, respectively [10]. Recent analyses and studies demonstrated the existence of stem cells in bone marrow and PDL by flow cytometric analysis [2, 3, 7, 10]. In the present study, a cell population, which shows stem cell features, was isolated from PDL while we made an effort to isolate MSCs from bone marrow. The expression of specific surface markers of stem cells, including CD90, CD105, CD166, and CD73, confirms stem cell isolation from the tibial bone marrow and PDL and the expression of CD44 and CD146 confirms the MSCs and PDLs isolation while hematopoietic stem cell markers such as CD45 and CD34 were not expressed. For growth, development, and continuous proliferation under *in vitro* conditions, BM-MSCs need factors associated with bone marrow, whereas PDLSCs are differentiated from the hard tissue of PDL and they are less dependent on these factors [9], this is one of the advantages of utilizing this type of stem cells in regenerative treatment and reconstruction of injured tissue. For this reason, PDLSCs were placed in differentiation medium, containing cytokines, whereas considering the previous studies, the differentiation procedure under *in vitro* conditions was completely predictable [9]. To ensure whether osteoblasts had been differentiated and formed or not, the activation procedure of calcium was traced with alizarin red staining. Red appearance of calcium deposition after staining indicated osteogenesis. Moreover, in order to confirm this matter, osteoblast-specific gene expression was evaluated by RT-PCR technique. One of the evaluated genes was Cbf1 of Runx family. The creation of this protein is essential for differentiation of the cells into osteoblasts [11]. The expression of this gene is thus a sign for osteoblast differentiation. Another analyzed gene was BGLAP. Osteocalcin gene indicates that it plays an important role in osteoblast differentiation and bone induction. Osteocalcin (BGLAP) is the most specific marker of osteoblasts, consequently the expression of genes and factors encoding for osteocalcin plays a fundamental role [12]. BMP4 is one of the members of TGF-β family; thus, it has a role in cells differentiation into the osteoblasts and chonroblasts like other subgroups of this family [2, 13]. In such case, the gene expression and creation of this protein confirm osteogenesis procedure and osteoblast differentiation. Although it has been indicated that PDLSCs have the potential to express osteoblast and cementoblast markers, forming the dentin, bone, and their associated hematopoietic parts appeared under *in vivo* conditions in the previous studies [9]. Bone is a mineralized connective tissue and carries out multiple functions. Osteoprogenitor cell differentiation is a vital process for osteogenesis. In this process, mesenchymal progenitor cells (MPCs) are differentiated into osteoblasts and osteogenesis is developed. In the first stage, progenitor cells proliferate by appropriate signals and the extracellular matrix secretion occurs; it then becomes mineralized and the cells will be embedded in the matrix. During cell differentiation, the genes are expressed within the cells and the resulting protein plays an important role in osteogenesis. DLX3 is one of these genes that is specifically expressed in all developing osteoblasts and osteocytes. This gene is essential for osteoblastic differentiation and skeletal morphogenesis. In addition, it acts as a scaffold for nucleic acids and regulatory factors involved in skeletal gene expression. Consequently, DLX3 gene expression can be a sign for cell differentiation into osteoblast.

The decreased BMP signaling in the ameloblasts and odontoblasts clearly implies reduced DLX3 gene expression in ameloblasts [14]. During tooth morphogenesis, DLX3 is first expressed in dental epithelium in which ameloblasts are differentiated for enamel formation and thereafter expressed in dental epithelial-mesenchymal (DE-DM) cells. The transcription factors in tooth root development are the members of homeobox gene family, including DLX2 and DLX3. In knocked out mice, deletion in DLX3 gene leads to no roots in the maxillary teeth, so there is no attachment between the teeth and the bone. Histological analysis indicates normal formation crown, enamel, dentine, formation of Hertwig’s epithelial root sheath (HERS) and its body, but no growth had occurred in the roots, cementum, and ligament. Therefore, the above gene expression is essential for the growth of periodontal ligament. Meanwhile, deletion and mutation in genes involved in tooth development such as DLX3 contributes to odontogenesis and amelogenesis, enlarged pulp chambers, less dentin in the crown and root, and also the enamel becomes thinner and hypominerilized. 

In the present study, calvarium bone regeneration has been investigated and shown *in vivo*. In this study, it has been tried to evaluate the regeneration and reconstruction procedures in calvarial defects using BM-MSCs, PDLSCs, and pre-osteoblasts, whereas the rate of healing and bone reconstruction were investigated by collagen membrane and also by adding collagen gel into osteoinductive medium. In microscopic analyses, the BM-MSCs-filled bone defect revealed a significant difference in osteoregeneration rate in comparison with control group and other three defects, which had been separately filled with PDLSCs, preosteoblasts, and collagen gel. Osteogenesis in the middle of the cavity or on the collagen membrane represented that collagen matrix is a suitable carrier and substrate for cells. Results showed that the stem cells and pre-osteoblasts remained alive and they proliferated and differentiated by transferring them to the defect. In comparison with the BM-MSCs- and PDLSCs-treated cases, it is suggested that the reason for advancing the reconstruction procedure can be ascribed to the potential of cells and the type of reconstructed tissue. BM-MSCs probably act more powerful, whereas PDLSCs were changed to the progenitor cells and they show slow differentiation into osteoblasts *in vivo *and finally gave rise to the reconstruction of calvarial defect. It is proposed that the bone type involves in stem cells differentiation, and this can be justifiable considering microenvironmental induction. With respect to the significant role of environmental induction and *in situ* signals in developing reconstruction procedure, it seems that these signals probably have little effect on PDLSCs in calvarium. In other words, it seems that the differentiation was progressed more slowly in calvarial bone by these cells considering the potential of PDLSCs and the results of the previous studies, whereas they are more effective in the reconstruction procedure of periodontium [15-17]. In order to give a reason for slow reconstruction procedure of bone treating with preosteoblasts, the minor effects of *in situ* inductive signals on differentiated cells can be implied. Therefore, it is proposed that these cells make the reconstruction process slower and subsequently the rate of osteogenesis will become lower. In collage memberane-treated samples and osteo-inductive medium containing cell suspension, the bone regeneration was slow in the middle of the cavity. For justifying slow bone reconstruction, it can be implied that the treated defects connected with periost and dura were not able to receive the environmental signals for reconstruction. In general, it can be concluded that PDLSCs as well as BM-MSCs have the differentiation potential into osteoblast [18-19]. With regard to the previous studies, these cells involve in the reconstruction procedure of periodontium, but the results of the present study suggest that the potential of this type of stem cell is less than BM-MSCs in reconstruction and regeneration of calvarial bone [20]. This was probably caused by different bone types and effective environmental signals in reconstruction. In fact, more differentiated cells give rise to the lower potential for reconstruction and regeneration. Therefore, the results of reconstruction rate in pre-osteoblast-treated defects represent this matter.
